# Supplementary material for: Dichotomy between extracellular signatures of active dendritic chemical synapses and gap junctions
Source: eLife. 2026 Jul 30;14:RP103046. doi: 10.7554/eLife.103046 (PMC13423354; doi:10.7554/eLife.103046)
Supplement: Peer review history

# Editor report: eLife Assessment

**DOI:** 10.7554/eLife.103046.3.sa0

**Authors:** Ching-Lung Hsu

**Affiliations:** National Taiwan University China

**Keywords:** Compelling, Valuable

This study presents a **valuable** theoretical exploration on the electrophysiological mechanisms of ionic currents via gap junctions in hippocampal CA1 pyramidal-cell models, and their potentially unique contribution to local field potentials (LFPs). The biophysical foundations of transmembrane electric dipoles, and the associated argument points, are generally **compelling**. Experimental constraints on gap junctions and strictly quantitative matching between chemical vs. junctional inputs have been hard to achieve. This computational investigation thus offers a specific way to enhance conceptual understanding and provides interesting testable predictions, which would be of great interest to experimental neurophysiologists who interpret relevant recordings.

# Referee report: Reviewer #1 (Public review)

**DOI:** 10.7554/eLife.103046.3.sa1

This manuscript makes a significant contribution to the field by exploring the dichotomy between chemical synaptic and gap junctional contributions to extracellular potentials. While the study is comprehensive in its computational approach, adding experimental validation, network-level simulations, and expanded discussion on implications would elevate its impact further.

Strengths:

Novelty and Scope:

The manuscript provides a detailed investigation into the contrasting extracellular field potential (EFP) signatures arising from chemical synapses and gap junctions, an underexplored area in neuroscience.

It highlights the critical role of active dendritic processes in shaping EFPs, pushing forward our understanding of how electrical and chemical synapses contribute differently to extracellular signals.

Methodological Rigor:

The use of morphologically and biophysically realistic computational models for CA1 pyramidal neurons ensures that the findings are grounded in physiological relevance.

Systematic analysis of various factors, including the presence of sodium, leak, and HCN channels, offers a clear dissection of how transmembrane currents shape EFPs.

Biological Relevance:

The findings emphasize the importance of incorporating gap junctional inputs in analyses of extracellular signals, which have traditionally focused on chemical synapses.

The observed polarity differences and spectral characteristics provide novel insights into how neural computations may differ based on the mode of synaptic input.

Clarity and Depth:

The manuscript is well-structured, with logical progression from synchronous input analyses to asynchronous and rhythmic inputs, ensuring comprehensive coverage of the topic.

Comments on revised version:

The authors have addressed all my concerns in the revised version of the manuscript.

# Referee report: Reviewer #2 (Public review)

**DOI:** 10.7554/eLife.103046.3.sa2

Summary:

This computational work examines whether the inputs that neurons receive through electrical synapses (gap junctions) have different signatures in the extracellular local field potential (LFP) compared to inputs via chemical synapses. The authors present the results of a series of model simulations where either electric or chemical synapses targeting a single hippocampal pyramidal neuron are activated in various spatio-temporal patterns, and the resulting LFP in the vicinity of the cell is calculated and analyzed. The authors find several notable qualitative differences between the LFP patterns evoked by gap junctions vs. chemical synapses. For some of these findings, the authors demonstrate convincingly that the observed differences are explained by the electric vs. chemical nature of the input, and these results likely generalize to other cell types. However, in other cases, it remains plausible (or even likely) that the differences are caused, at least partly, by other factors (such as different intracellular voltage responses due to differences in the amplitudes and time courses of the input currents). Furthermore, it was not immediately clear to me how the results could be applied to analyze more realistic situations where neurons receive partially synchronized excitatory and inhibitory inputs via chemical and electric synapses.

Strengths:

The main strength of the paper is that it draws attention to the fact that inputs to a neuron via gap junctions are expected to give rise to a different extracellular electric field compared to inputs via chemical synapses, even if the intracellular effects of the two types of input are similar. This is because, unlike chemical synaptic inputs, inputs via gap junctions are not directly associated with transmembrane currents. This is a general result that holds independent of many details such as the cell types or neurotransmitters involved.

Another strength of the article is that the authors attempt to provide intuitive, non-technical explanations of most of their findings, which should make the paper readable also for non-expert audiences (including experimentalists).

Weaknesses:

The most problematic aspect of the paper relates to the methodology for comparing the effects of electric vs. chemical synaptic inputs on the LFP. The authors seem to suggest that the primary cause of all the differences seen in the various simulation experiments is the different nature of the input, and particularly the difference between the transmembrane current evoked by chemical synapses and the gap junctional current that does not involve the extracellular space. However, this is clearly an oversimplification: since no real attempt is made to quantitatively match the two conditions that are compared (e.g., regarding the strength and temporal profile of the inputs), the differences seen can be due to factors other than the electric vs. chemical nature of synapses. In fact, if inputs were identical in all parameters other than the transmembrane vs. directly injected nature of the current, the intracellular voltage responses and, consequently, the currents through voltage-gated and leak currents would also be the same, and the LFPs would differ exactly by the contribution of the transmembrane current evoked by the chemical synapse. This is evidently not the case for any of the simulated comparisons presented, and the differences in the membrane potential response are rather striking in several cases (e.g., in the case of random inputs, there is only one action potential with gap junctions, but multiple action potentials with chemical synapses). Consequently, it remains unclear which observed differences are fundamental in the sense that they are directly related to the electric vs. chemical nature of the input, and which differences can be attributed to other factors such as differences in the strength and pattern of the inputs (and the resulting difference in the neuronal electric response).

Some of the explanations offered for the effects of cellular manipulations on the LFP appear to be incomplete. More specifically, the authors observed that blocking leak channels significantly changed the shape of the LFP response to synchronous synaptic inputs - but only when electric inputs were used, and when sodium channels were intact. The authors seemed to attribute this phenomenon to a direct effect of leak currents on the extracellular potential - however, this appears unlikely both because it does not explain why blocking the leak conductance had no effect in the other cases, and because the leak current is several orders of magnitude smaller than the spike-generating currents that make the largest contributions to the LFP. An indirect effect mediated by interactions of the leak current with some voltage-gated currents appears to be the most likely explanation, but identifying the exact mechanism would require further simulation experiments and/or a detailed analysis of intracellular currents and the membrane potential in time and space.

In every simulation experiment in this study, inputs through electric synapses are modeled as intracellular current injections of pre-determined amplitude and time course based on the sampled dendritic voltage of potential synaptic partners. This is a major simplification that may have a significant impact on the results. First, the current through gap junctions depends on the voltage difference between the two connected cellular compartments and is thus sensitive to the membrane potential of the cell that is treated as the neuron "receiving" the input in this study (although, strictly speaking, there is no pre- or postsynaptic neuron in interactions mediated by gap junctions). This dependence on the membrane potential of the target neuron is completely missing here. A related second point is that gap junctions also change the apparent membrane resistance of the neurons they connect, effectively acting as additional shunting (or leak) conductance in the relevant compartments. This effect is completely missed by treating gap junctions as pure current sources.

One prominent claim of the article that is emphasized even in the abstract is that HCN channels mediate an outward current in certain cases. Although this statement is technically correct, there are two reasons why I do not consider this a major finding of the paper. First, as the authors acknowledge, this is a trivial consequence of the relatively slow kinetics of HCN channels: when at least some of the channels are open, any input that is sufficiently fast and strong to take the membrane potential across the reversal potential of the channel will lead to the reversal of the polarity of the current. This effect is quite generic and well-known, and is by no means specific to gap junctional inputs or even HCN channels. Second, and perhaps more importantly, the functional consequence of this reversed current through HCN channels is likely to be negligible. As clearly shown in Supplementary Figure S4, the HCN current becomes outward only for an extremely short time period during the action potential, which is also a period when several other currents are also active and likely dominant due to their much higher conductances. I also note that several of these relevant facts remain hidden in Figure 3, both because of its focus on peak values, and because of the radically different units on the vertical axes of the current plots.

Finally, I missed an appropriate validation of the neuronal model used, and also the characterization of the effects of the in silico manipulations used on the basic behavior of the model. As far as I understand, the model in its current form has not been used in other studies, although it is closely related to models used in earlier modeling work from the same laboratory. If this is the case, it would be important to demonstrate convincingly through (preferably quantitative) comparisons with experimental data using different protocols that the model captures the physiological behavior of at least the relevant compartments (in this case, the dendrites and the soma) of hippocampal pyramidal neurons sufficiently well that the results of the modeling study are relevant to the real biological system. In addition, the correct interpretation of various manipulations of the model would be strongly facilitated by investigating and discussing how the physiological properties of the model neuron are affected by these alterations.

Comments on revised version:

The authors made mainly cosmetic changes in the manuscript (primarily by adding more discussion), and most of these do not affect my earlier assessment. I have updated my Public Review in a few places to reflect those few changes that substantially address my previous concerns.

# Author comment: Author response

**DOI:** 10.7554/eLife.103046.3.sa3

**Authors:** Richa Sirmaur, Rishikesh Narayanan

**Affiliations:** Indian Institute of Science Bangalore Bangalore India; Indian Institute of Science Bangalore Bangalore India

The following is the authors’ response to the original reviews.

**eLife Assessment:**

This study presents a valuable theoretical exploration on the electrophysiological mechanisms of ionic currents via gap junctions in hippocampal CA1 pyramidal-cell models, and their potential contribution to local field potentials (LFPs) that is different from the contribution of chemical synapses. The biophysical argument regarding electric dipoles appears solid, but the evidence can be more convincing if their predictions are tested against experiments. A shortage of model validation and strictly comparable parameters used in the comparisons between chemical vs. junctional inputs makes the modeling approach incomplete; once strengthened, the finding can be of broad interest to electrophysiologists, who often make recordings from regions of neurons interconnected with gap junctions.

We gratefully thank the editors and the reviewers for the time and effort in rigorously assessing our manuscript, for the constructive review process, for their enthusiastic responses to our study, and for the encouraging and thoughtful comments. We especially thank you for deeming our study to be a valuable exploration on the differential contributions of active dendritic gap junctions vs. chemical synapses to local field potentials. We thank you for your appreciation of the quantitative biophysical demonstration on the differences in electric dipoles that appear in extracellular potentials with gap junctions vs. chemical synapses.

However, we are surprised by aspects of the assessment that resulted in deeming the approach incomplete, especially given the following with specific reference to the points raised:

(1) Testing against experiments: With specific reference to gap junctions, quantitative experimental verification becomes extremely difficult because of the well-established non specificities associated with gap junctional modulators (Behrens et al., 2011; Rouach et al., 2003). In addition, genetic knockouts of gap junctional proteins are either lethal or involve functional compensation (Bedner et al., 2012; Lo, 1999), together making causal links to specific gap junctional contributions with currently available techniques infeasible.

In addition, the complex interactions between co-existing chemical synaptic, gap junctional, and active dendritic contributions from several cell-types make the delineation of the contributions of specific components infeasible with experimental approaches. A computational approach is the only quantitative route to specifically delineate the contributions of individual components to extracellular potentials, as seen from studies that have addressed the question of active dendritic contributions to field potentials (Halnes et al., 2024; Ness et al., 2018; Reimann et al., 2013; Sinha & Narayanan, 2015, 2022) or spiking contributions to local field potentials (Buzsaki et al., 2012; Gold et al., 2006; Schomburg et al., 2012). The biophysically and morphologically realistic computational modeling route is therefore invaluable in assessing the impact of individual components to extracellular field potentials (Einevoll et al., 2019; Halnes et al., 2024).

Together, we emphasize that the computational modeling route is currently the only quantitative methodology to delineate the contributions of gap junctions vs. chemical synapses to extracellular potentials.

(2) Model validation: The model used in this study was adopted from a physiologically validated model from our laboratory (Roy & Narayanan, 2021). Please note that the original model was validated against several physiological measurements along the somatodendritic axis. We sincerely regret our oversight in not mentioning clearly that we have used an existing, thoroughly physiologically-validated model from our laboratory in this study.

(3) Comparisons between chemical vs. junctional inputs: We had taken elaborate precautions in our experimental design to match the intracellular electrophysiological signatures with reference to synchronous as well as oscillatory inputs, irrespective of whether inputs arrived through gap junctions or chemical synapses. A new Supplementary Figure S3 has been added to address this concern raised by the reviewers.

In the revised manuscript, we have addressed all the concerns raised by the reviewers in detail. We have provided point-by-point responses to reviewers’ helpful and constructive comments below. We thank the editors and the reviewers for this constructive review process, which helped us in improving our manuscript with specific reference to emphasizing the novelty of our approach and conclusions. The specific changes incorporated into the revised manuscript are detailed below.

**Reviewer #1 (Public review):**

This manuscript makes a significant contribution to the field by exploring the dichotomy between chemical synaptic and gap junctional contributions to extracellular potentials. While the study is comprehensive in its computational approach, adding experimental validation, network-level simulations, and expanded discussion on implications would elevate its impact further.

We gratefully thank you for your time and effort in rigorously assessing our manuscript, for the enthusiastic response, and the encouraging and thoughtful comments on our study. In what follows, we have provided point-by-point responses to the specific comments.

Strengths

Novelty and Scope

The manuscript provides a detailed investigation into the contrasting extracellular field potential (EFP) signatures arising from chemical synapses and gap junctions, an underexplored area in neuroscience. It highlights the critical role of active dendritic processes in shaping EFPs, pushing forward our understanding of how electrical and chemical synapses contribute differently to extracellular signals.

We thank you for the positive comments on the novelty of our approach and how our study addresses an underexplored area in neuroscience. The assumptions about the passive nature of dendritic structures had indeed resulted in an underestimation of the contributions of gap junctions to extracellular potentials. Once the realities of active structures are accounted for, the contributions of gap junctions increases by several orders of magnitude compared to passive structures (Fig. 1D).

Methodological Rigor

The use of morphologically and biophysically realistic computational models for CA1 pyramidal neurons ensures that the findings are grounded in physiological relevance. Systematic analysis of various factors, including the presence of sodium, leak, and HCN channels, offers a clear dissection of how transmembrane currents shape EFPs.

We thank you for your encouraging comments on the experimental design and methodological rigor of our approach.

Biological Relevance

The findings emphasize the importance of incorporating gap junctional inputs in analyses of extracellular signals, which have traditionally focused on chemical synapses. The observed polarity differences and spectral characteristics provide novel insights into how neural computations may differ based on the mode of synaptic input.

We thank you for your positive comments on the biological relevance of our approach. We also gratefully thank you for emphasizing the two striking novelties unveiling the dichotomy between gap junctions and chemical synapses in their contributions to field potentials: polarity differences and spectral characteristics.

Clarity and Depth

The manuscript is well-structured, with a logical progression from synchronous input analyses to asynchronous and rhythmic inputs, ensuring comprehensive coverage of the topic.

We sincerely thank you for the positive comments on the structure and comprehensive coverage of our manuscript encompassing different types of inputs that neurons typically receive.

Weaknesses and Areas for Improvement

Generality and Validation

The study focuses exclusively on CA1 pyramidal neurons. Expanding the analysis to other cell types, such as interneurons or glial cells, would enhance the generalizability of the findings. Experimental validation of the computational predictions is entirely absent. Empirical data correlating the modeled EFPs with actual recordings would strengthen the claims.

We thank you for raising this important point. The prime novelty and the principal conclusion of this study is that gap junctional contributions to extracellular field potentials are orders of magnitude higher when the active nature of cellular compartments are accounted for. The lacuna in the literature has been consequent to the assumption that cellular compartments are passive, resulting in the dogma that gap junctional contributions to field potentials are negligible. Despite knowledge about active dendritic structures for decades now, this assumption has kept studies from understanding or even exploring the contributions of gap junctions to field potentials. The rationale behind the choice of a computational approach to address the lacuna were as follows:

(1) The complex interactions between co-existing chemical synaptic, gap junctional, and active dendritic contributions from several cell-types make the delineation of the contributions of specific components infeasible with experimental approaches. A computational approach is the only quantitative route to specifically delineate the contributions of individual components to extracellular potentials, as seen from studies that have addressed the question of active dendritic contributions to field potentials (Halnes et al., 2024; Ness et al., 2018; Reimann et al., 2013; Sinha & Narayanan, 2015, 2022) or spiking contributions to local field potentials (Buzsaki et al., 2012; Gold et al., 2006; Schomburg et al., 2012). The biophysically and morphologically realistic computational modeling route is therefore invaluable in assessing the impact of individual components to extracellular field potentials (Einevoll et al., 2019; Halnes et al., 2024).

(2) With specific reference to gap junctions, quantitative experimental verification becomes extremely difficult because of the well-established non-specificities associated with gap junctional modulators (Behrens et al., 2011; Rouach et al., 2003). 'The non-specific actions of gap junctions are tabulated in Table 2 of (Szarka et al., 2021). In addition, genetic knockouts of gap junctional proteins are either lethal or involve functional compensation (Bedner et al., 2012; Lo, 1999), together making causal links to specific gap junctional contributions with currently available techniques infeasible.

We highlight the novelty of our approach and of the conclusions about differences in extracellular signatures associated with active-dendritic chemical synapses and gap junctions, against these experimental difficulties. We emphasize that the computational modeling route is currently the only quantitative methodology to delineate the contributions of gap junctions vs. chemical synapses to extracellular potentials. Our analyses clearly demonstrates that gap junctions do contribute to extracellular potentials if the active nature of the cellular compartments is explicitly accounted for (Fig. 1D). We also show theoretically well-grounded and mechanistically elucidated differences in polarity (Figs. 1–3) as well as in spectral signatures (Figs. 5–8) of extracellular potentials associated with gap junctional vs. chemical synaptic inputs. Together, our fundamental demonstration in this study is the critical need to account for the active nature of cellular compartments in studying gap junctional contributions of extracellular potentials, with CA1 pyramidal neuronal dendrites used as an exemplar.

In the revised version of the manuscript, we have emphasized the motivations for the approach we took, highlighting the specific novelties both in methodological and conceptual aspects, finally emphasizing the need to account for other cell types and gap junctional contributions therein. Importantly, we have emphasized the non-specificities associated with gap-junctional blockers as the reason why experimental delineation of gap junctional vs. chemical synaptic contributions to LFP becomes tedious. We believe that these points underscore the need for the computational approach that we took to address this important question, apart from the novelties of the study.

In response to your constructive comments, we have added the following to the revised version of the manuscript, in the Introduction section as motivation for the specific route we took:

“Given the complexity arising from the concurrent activity of chemical synapses, gap junctions, and active dendritic conductances across multiple neuronal populations, experimentally isolating the contributions of individual components to extracellular potentials remains highly challenging. To address this limitation, we employed a computational modeling approach, which provides a quantitative framework for systematically dissecting the distinct roles of specific cellular and synaptic elements. This strategy is consistent with previous studies that have successfully used computational methods to elucidate the contributions of active dendritic mechanisms to LFPs (Halnes et al., 2024; Ness et al., 2018; Reimann et al., 2013; Sinha & Narayanan, 2015, 2022) or spiking contributions to LFPs (Buzsaki et al., 2012; Gold et al., 2006; Schomburg et al., 2012). In addition, experimentally isolating the contribution of gap junctions is complicated by non-specific effects of available pharmacological modulators targeting these connections (Behrens et al., 2011; Rouach et al., 2003). Most genetic knockouts of gap junctional proteins are either lethal or trigger functional compensatory mechanisms (Bedner et al., 2012; Lo, 1999), thereby rendering causal attribution of specific gap junctional contributions infeasible with currently available experimental approaches. Consequently, biophysically and morphologically detailed computational modeling provides a crucial means to evaluate the impact of individual neuronal components on extracellular field potentials (Einevoll et al., 2019; Halnes et al., 2024).”

We thank you for raising this point as this allowed us to expand on the specific motivations for the approach we took, and to present the specific novelties of our study to the analyses of extracellular field potentials. Thank you.

Role of Active Dendritic Currents

The paper emphasizes active dendritic currents, particularly the role of HCN channels in generating outward currents under certain conditions. However, further discussion of how this mechanism integrates into broader network dynamics is warranted.

We thank you for this constructive suggestion. We agree that it is important to consider the implications for broader network dynamics of the outward HCN currents that are observed with synchronous inputs. In the revised manuscript, we have elaborated on the implications of the outward HCN current to network dynamics in detail. The following paragraph has been added to Discussion subsection on “Outward HCN currents regulate extracellular potentials”:

“HCN channels play a critical role in shaping hippocampal network dynamics by modulating neuronal excitability, oscillatory behavior, and susceptibility to pathological states (Kessi et al., 2022; Magee, 1998; Mishra & Narayanan, 2025; Nolan et al., 2004). The outward-like properties of the HCN current we observed may have specific functional implications at different scales. At the cellular scale, the manifestation of outward current during action potentials or plateau potentials could contribute to after hyperpolarization thereby regulating firing properties. In cortical and hippocampal pyramidal neurons, most single-neuron processing occurs in their elaborate dendritic branches, where there is spatiotemporal summation of different synaptic potentials, plateau potentials, back propagating action potentials, and dendritic spikes (Johnston & Narayanan, 2008; Major et al., 2013; Stuart & Spruston, 2015). Considering the heavy expression of HCN channels in the dendrites of hippocampal and cortical pyramidal neurons (Kole et al., 2006; Lorincz et al., 2002; Magee, 1998; Williams & Stuart, 2000), the back propagating action potentials, plateau potentials, or dendritic spikes at dendritic location could yield outward currents. These outward currents could act as a hyperpolarizing mechanism that suppresses spatiotemporal summation of the different dendritic potentials.

At the network scale, such regulation of dendritic potentials and somatic firing could contribute to overall reduction in firing rates of different neurons in the network. For instance, as inhibitory neurons typically elicit action potentials at higher frequencies, somatic outward HCN currents would occur more frequently in inhibitory neurons that express HCN channels compared to excitatory neurons. However, the heavy expression of HCN channels in the dendrites and the higher prevalence of dendritic spikes and plateau potentials in dendrites (Basak & Narayanan, 2018; Larkum et al., 2022; Moore et al., 2017) imply that the impact on outward HCN currents might be higher. Thus, the presence of outward HCN currents would regulate network balance of excitation inhibition in an activity-dependent manner. Additionally, the outward component of the current through HCN channels could contribute to stabilization of network synchrony by promoting spike phase coherence and to modulation of spike-LFP phase relationships (Das et al., 2017; Ness et al., 2016, 2018; Seenivasan & Narayanan, 2020; Sinha & Narayanan, 2015, 2022).

Together, the outward HCN current could play critical roles in regulating several cellular and network functions including spatiotemporal summation within single neurons, amplitude and phase of different oscillations, excitatory-inhibitory interactions, and rate and temporal coding involved in spatial navigation (Hussaini et al., 2011; Nolan et al., 2004; O'Keefe & Recce, 1993). In the context of brain rhythms, future investigations are needed to explore ripple-frequency oscillations, specifically to assess whether high-frequency network interactions are modulated by HCN outward currents. Importantly, future studies could specifically focus on delineating the prevalence and specific contributions of outward currents through HCN channels to single-neuron and network physiology.”

We thank you for highlighting this point, as it allowed us to elaborate the broader roles of HCN channels to single-cell computation, network dynamics, and field potentials. Thank you.

Analysis of Plasticity

While the manuscript mentions plasticity in the discussion, there are no simulations that account for activity-dependent changes in synaptic or gap junctional properties. Including such analyses could significantly enhance the relevance of the findings.

We thank you for this constructive suggestion. Please note that we have presented consistent results for both fewer and more gap junctions in our analyses (Figure 1 with 217 gap junctions and Supplementary Figure 1 with 99 gap junctions). Thus, our fundamentally novel result that gap junctions onto active dendrites differentially shape LFPs holds true irrespective of the relative density of gap junctions onto the neuron. Thus, these results demonstrate that the conclusions about their contributions to LFP are invariant to plasticity in their gap junctional numerosity.

We had only briefly mentioned plasticity in the Introduction to highlight the different modes of synaptic transmission and to emphasize that plasticity has been studied in both chemical synapses and gap junctions, playing a role in learning and adaptation. However, it seems that this wording inadvertently suggested that our study includes plasticity simulations. Therefore, we have removed that sentence from Introduction in the revised manuscript to ensure clarity.

In the ‘Limitations of analyses and future studies’ section in Discussion, we suggested investigating the impact of plasticity mechanisms—specifically, activity-dependent plasticity of ion channels—on synaptic receptors vs. gap junctions and their effects on extracellular field potentials under various input conditions and plasticity combinations across different structures. We fully agree with the reviewer that such studies would offer valuable insights and further enhance the broader relevance of our findings. However, while our study implies this direction, it was not the primary focus of our investigation.

In the revised manuscript, we have also expanded on intrinsic/synaptic plasticity and how they could contribute to LFPs (Sinha & Narayanan, 2015, 2022), while also pointing to simulations with different numbers of gap junction in this context. The following specific changes have been incorporated to the revised manuscript:

Discussion subsection “Limitations of analyses and future directions”

“We demonstrated that the contribution of gap junctions to extracellular field potentials remains consistent regardless of the number of gap junctions. Specifically, we showed that the distinct positive LFP deflections persisted irrespective of their relative density on neurons (Fig. 1 with 217 gap junctions and Supplementary Fig. 1 with 99 gap junctions). Previous studies have quantitatively demonstrated that intrinsic and synaptic plasticity modulate hippocampal LFPs and phase coding (Sinha & Narayanan, 2015, 2022). Future analyses should also assess the impact of activity-dependent plasticity in ion channels (on dendrites, axonal initial segments, and other compartments), in synaptic receptors, and in gap junctions (Andersen et al., 2006; Coulon & Landisman, 2017; Johnston & Narayanan, 2008; Magee & Grienberger, 2020; Mishra & Narayanan, 2021; Neves et al., 2008; O'Brien, 2014; Pereda, 2014; Vaughn & Haas, 2022) on extracellular potentials with various kinds of gap junctional inputs and different combinations of plasticity in various structures. Interactions among different forms of plasticity and how co-dependent plasticity in different components alters extracellular field potentials could provide deeper insights about physiological changes during learning and pathological changes observed in different neurological disorders (Sinha & Narayanan, 2022).”

We thank you for highlighting this as this allowed us to improve on the specific focus of the manuscript and the study. Thank you.

Frequency-Dependent Effects

The study demonstrates that gap junctional inputs suppress highfrequency EFP power due to membrane filtering. However, it could delve deeper into the implications of this for different brain rhythms, such as gamma or ripple oscillations.

We sincerely thank you for these insightful comments that we totally agree with. As it so happens, this manuscript forms the first part of a broader study where we explore the implications of gap junctions to ripple frequency oscillations. The ripple oscillations part of the work was presented as a poster in the Society for Neuroscience (SfN) annual meeting 2024 (Sirmaur & Narayanan, 2024). There, we simulate a neuropil made of hundreds of morphologically realistic neurons to assess the role of different synaptic inputs excitatory, inhibitory, and gap junctional and active dendrites to ripple frequency oscillations. We demonstrate there that the conclusions from single-neuron simulations in this current manuscript extend to a neuropil with several neurons, each receiving excitatory, inhibitory and gap-junctional inputs, especially with reference to high-frequency oscillations. Our network based analyses unveiled a dominant mediatory role of patterned inhibition in ripple generation, with recurrent excitations through chemical synapses and gap junctions in conjunction with return-current contributions from active dendrites playing regulatory roles in determining ripple characteristics (Sirmaur & Narayanan, 2024).

Our principal goal in this study, therefore, was to lay the single-neuron foundation for network analyses of the impact of gap junctions on LFPs. We are preparing the network part of the study, with a strong focus on ripple-frequency oscillations, for submission for peer review separately. Please see abstract of our poster presented at the Society for Neuroscience annual meeting 2024 on the topic here: https://tinyurl.com/57ehvsep.

In the revised manuscript, we have mentioned the results from our SfN abstract with reference to network simulations and high-frequency oscillations, while also presenting discussions from other studies on the role of gap junctions in synchrony and LFP oscillations. The following has been added to the revised manuscript under the Discussion subsection “High-frequency LFP power was suppressed with gap junctional inputs”:

“In this context, our analyses lay the foundation for network analyses of the impact of gap junctions on LFPs. The conclusions from the single-neuron simulations in this study extend to a neuropil with several neurons, each receiving synaptic and gap junctional inputs, especially with reference to high-frequency ripple oscillations (Sirmaur & Narayanan, 2024). A neuropil made of hundreds of morphologically realistic pyramidal neurons was used to assess the role of different synaptic inputs excitatory, inhibitory, and gap junctional with different patterns of stimulation and active dendritic contributions to ripple-frequency oscillations. Network-based analyses have unveiled a dominant mediatory role of patterned inhibition in ripple generation, with recurrent excitations through chemical synapses and gap junctions, in conjunction with return-current contributions from active dendrites, playing modulatory roles in governing ripple characteristics (Sirmaur & Narayanan, 2024). Future studies could expand on these conclusions to explore the implications of frequency-dependent filtering (with reference to gap junctional coupling) on high-frequency extracellular oscillations.”

We thank you for highlighting this point as it allowed us to expand on the implications for our analyses to brain rhythms, especially with reference to high-frequency oscillations. Thank you.

Visualization

Figures are dense and could benefit from more intuitive labeling and focused presentations. For example, isolating key differences between chemical and gap junctional inputs in distinct panels would improve clarity.

We thank you for this constructive suggestion. We used the specific visualization throughout, where we place the outcomes associated with chemical synapses and gap junctions in the same figure, adjacent to each other. We believe that this offers visually intuitive distinction between the outcomes for chemical synapses and gap junctions, rather than placing them in different figures. Splitting them would place the outcomes in different figures and requires turning pages or placing two different figures adjacent to each other for quantitative comparison. We respectfully request that we be allowed to retain this form of visualization in the figures. Thank you.

Contextual Relevance

The manuscript touches on how these findings relate to known physiological roles of gap junctions (e.g., in gamma rhythms) but does not explore this in depth. Stronger integration of the results into known neural network dynamics would enhance its impact.

We sincerely appreciate your valuable suggestion and acknowledge the importance of integrating our results into established neural network dynamics, particularly their implications for gamma rhythms. We have addressed this aspect in the revised version of our manuscript. We have added this to the Discussion subsection on “High-frequency LFP power was suppressed with gap junctional inputs” of the revised manuscript:

“In the context of oscillations and gap-junctional coupling, electrical synapses have been shown to regulate the emergence and stability of the network interactions underlying rhythms of different frequencies, especially gamma-frequency oscillations (Bocian et al., 2009; Buhl et al., 2003; Draguhn et al., 1998; Hormuzdi et al., 2001; Konopacki et al., 2004; LeBeau et al., 2003; Posluszny, 2014; Traub et al., 2003). Specifically, both genetic and pharmacological manipulations of gap junctions have been shown to disrupt gamma rhythms. Genetic deletion of connexin-36 impairs the gamma oscillations associated with awake, active behavioral states (Buhl et al., 2003; Hormuzdi et al., 2001). High-frequency oscillations in the hippocampus have been shown to be sensitive to pharmacological agents like carbenoxolone and octanol that are known to inhibit gap junctions. Carbenoxolone has been known to reduce the transient gamma-frequency oscillations while octanol abolishes the persistent gamma rhythm (Draguhn et al., 1998; Hormuzdi et al., 2001; Posluszny, 2014; Traub et al., 2003). In the context of our results, where we demonstrate that the relative contributions of gap-junctional coupling to high-frequency extracellular potentials is low (Figs. 6–7), how do gap junctions contribute to enhanced extracellular gamma oscillations in these circuits?

It should be noted that in hippocampal circuits, gamma oscillations emerge predominantly due to interactions between inhibitory interneurons through GABAA103046 receptors (Buzsaki & Wang, 2012; Colgin, 2016; Colgin & Moser, 2010; Wang, 2010; Wang & Buzsaki, 1996; Whittington et al., 1995). Thus, the presence of additional gap junctional coupling between these inhibitory neurons allows for tighter synchrony between these reciprocally inhibition-coupled neurons. In other words, the presence of gap junctions increases the probability of action potential generation in other neurons that are electrically coupled to them, together increasing the population of inhibitory neurons that elicit synchronous action potentials. When these synchronous action potentials act on the adjacent cells, both excitatory and inhibitory, the transmembrane GABAA receptor currents yield stronger gamma-frequency oscillations in the extracellular potentials (Draguhn et al., 1998; Hormuzdi et al., 2001; Posluszny, 2014; Traub et al., 2003). Thus, the stronger high-frequency oscillations observed in these scenarios is owing to the enhanced synchrony that is brought about the gap-junctional coupling, which translates to stronger transmembrane inhibitory receptor currents.

These observations also strongly emphasize the utility of the computational approach we took in this study towards discerning the specific roles of gap junctions. Gap junctional coupling have strong physiological roles in terms of enhancing synchronous activity across the neurons that they couple and often express along with other receptors that connect the sets of neurons. Thus, the specific contributions of different neuronal components need to be studied with reference to how they contribute to physiological characteristics vs. their contributions to extracellular potentials. Thus, computational modeling offers an ideal route to understand the specific contributions of different neural-circuit components to extracellular field potentials and rhythms therein (Buzsaki et al., 2012; Einevoll et al., 2019; Einevoll et al., 2013; Sinha & Narayanan, 2022).”

We thank you for highlighting this point as this allowed us to delineate the impact of gap junctions to regulating synchrony across connected neurons vs. modulating field potentials. Thank you.

**Reviewer #2 (Public review):**

This computational work examines whether the inputs that neurons receive through electrical synapses (gap junctions) have different signatures in the extracellular local field potential (LFP) compared to inputs via chemical synapses. The authors present the results of a series of model simulations where either electric or chemical synapses targeting a single hippocampal pyramidal neuron are activated in various spatio-temporal patterns, and the resulting LFP in the vicinity of the cell is calculated and analyzed. The authors find several notable qualitative differences between the LFP patterns evoked by gap junctions vs. chemical synapses. For some of these findings, the authors demonstrate convincingly that the observed differences are explained by the electric vs. chemical nature of the input, and these results likely generalize to other cell types. However, in other cases, it remains plausible (or even likely) that the differences are caused, at least partly, by other factors (such as different intracellular voltage responses due to, e.g., the unequal strengths of the inputs). Furthermore, it was not immediately clear to me how the results could be applied to analyze more realistic situations where neurons receive partially synchronized excitatory and inhibitory inputs via chemical and electric synapses.

We gratefully thank you for your time and effort in rigorously assessing our manuscript, for the enthusiastic response, and the encouraging and thoughtful comments on our study. In what follows, we have provided point-by-point responses to the specific comments.

Strengths

The main strength of the paper is that it draws attention to the fact that inputs to a neuron via gap junctions are expected to give rise to a different extracellular electric field compared to inputs via chemical synapses, even if the intracellular effects of the two types of input are similar. This is because, unlike chemical synaptic inputs, inputs via gap junctions are not directly associated with transmembrane currents. This is a general result that holds independent of many details such as the cell types or neurotransmitters involved.

We gratefully thank you for the positive comments and the encouraging words about the novel contributions of our study. We are particularly thankful to you for your comment on the generality of our conclusions that hold for different cell types and neurotransmitters involved.

Another strength of the article is that the authors attempt to provide intuitive, non-technical explanations of most of their findings, which should make the paper readable also for non-expert audiences (including experimentalists).

We sincerely thank you for the positive comments about the readability of the paper.

Weaknesses

The most problematic aspect of the paper relates to the methodology for comparing the effects of electric vs. chemical synaptic inputs on the LFP. The authors seem to suggest that the primary cause of all the differences seen in the various simulation experiments is the different nature of the input, and particularly the difference between the transmembrane current evoked by chemical synapses and the gap junctional current that does not involve the extracellular space. However, this is clearly an oversimplification: since no real attempt is made to quantitatively match the two conditions that are compared (e.g., regarding the strength and temporal profile of the inputs), the differences seen can be due to factors other than the electric vs. chemical nature of synapses. In fact, if inputs were identical in all parameters other than the transmembrane vs. directly injected nature of the current, the intracellular voltage responses and, consequently, the currents through voltage-gated and leak currents would also be the same, and the LFPs would differ exactly by the contribution of the transmembrane current evoked by the chemical synapse. This is evidently not the case for any of the simulated comparisons presented, and the differences in the membrane potential response are rather striking in several cases (e.g., in the case of random inputs, there is only one action potential with gap junctions, but multiple action potentials with chemical synapses). Consequently, it remains unclear which observed differences are fundamental in the sense that they are directly related to the electric vs. chemical nature of the input, and which differences can be attributed to other factors such as differences in the strength and pattern of the inputs (and the resulting difference in the neuronal electric response).

We thank you for raising this important point. We would like to emphasize that our experimental design and analyses quantitatively account for the spatial distribution and temporal pattern of specific kinds of inputs that arrive through gap junctions and chemical synapses. We submit that our analyses quantitatively demonstrates that the fundamental difference between the gap junctional and chemical synaptic contributions to extracellular potentials is the absence of the direct transmembrane component from gap junctional inputs. We elucidate these points below:

(1) Spatial distribution: The inputs were distributed randomly across the basal dendrites, irrespective of whether they were through gap junctions or chemical synapses. For both chemical synapses and gap junctions, the inputs were of the same nature: excitatory.

(2) Different numbers of inputs: We have presented consistent results for both fewer and more gap junctions or chemical synapses in our analyses (see Figure 1 with 217 gap junctions or 245 chemical synapses and Supplementary Figure 2 with 99 gap junctions or 30 chemical synapses). Our fundamentally novel result that gap junctions onto active dendrites shape LFPs holds true irrespective of the relative density of gap junctions onto the neuron.

(3) Synchronous inputs (Figs. 1–3): For chemical synapses, the waveforms are in the shape of postsynaptic potentials. For gap junctional inputs, the waveforms are in the shape of postsynaptic potentials or dendritic spikes (to respect the active nature of inputs from the other cell). Here, the electrical response of the postsynaptic cell is identical irrespective of whether inputs arrive through gap junctions or chemical synapses: an action potential. We quantitatively matched the strengths such that the model generated a single action potential in response to synchronous inputs, irrespective of whether they arrived through chemical synaptic and gap junctional inputs. We mechanistically analyzed the contributions of different cellular components and show that the direct transmembrane current in chemical synapses is the distinguishing factor that determines the dichotomy between the contributions of gap junctions vs. chemical synapses to extracellular potentials (Figs. 2–3). In the revised manuscript, we have shown the intracellular responses to demonstrate that they are electrically matched (new Supplementary Figure 3).

(4) Random inputs (Fig. 4): For random inputs, we did not account for the number of action potentials that arrived, as the only observation we made here was with reference to the biphasic nature of the extracellular potentials with gap junctional inputs in the “No Sodium” scenario. We note that in the “No Sodium” scenario, the time-domain amplitudes were comparable for the field potentials (Fig. 4B, Fig. 4D).

(5) Rhythmic inputs (Fig. 5–8): For rhythmic inputs, please note that the intracellular and extracellular waveforms for every frequency are provided in supplementary figures S5– S11. It may be noted that the intracellular responses are comparable. In simulations for assessing spike-LFP comparison, we tuned the strengths to produce a single spike per cycle, ensuring fair comparison of LFPs with gap junctions vs. chemical synapses.

Taken together, we demonstrate through explicit sets of simulations and analyses that the differences in LFPs were not driven by the strength or patterns of the inputs but rather by the differences in direct transmembrane currents, which are subsequently reflected in the LFPs. In the revised manuscript, we have emphasized these points in the Discussion section, apart from providing intracellular traces for cases where they were not provided before (new Supplementary Figure 3):

Discussion subsection “Dominance of active dendritic currents with LFP associated with gap junctions”

“Our analyses quantitatively demonstrates that the fundamental difference between the gap junctional and chemical synaptic contributions to extracellular potentials is the absence of the direct transmembrane component from gap junctional inputs. A multitude of factors suggests that the observed LFP differences result not from variations in input strength or patterns but rather from differences in direct transmembrane currents, which are subsequently reflected in the LFP signals.

First, the inputs were distributed randomly across the basal dendrites, irrespective of whether they were through gap junctions or chemical synapses. For both chemical synapses and gap junctions, the inputs were exclusively excitatory in nature.

Second, the results remained consistent regardless of the number of gap junctions or chemical synapses. (Fig. 1 with 217 gap junctions or 245 chemical synapses and Supplementary Fig. 2 with 99 gap junctions or 30 chemical synapses). Our fundamentally novel result that gap junctions onto active dendrites shape LFPs holds true irrespective of the relative density of gap junctions onto the neuron.

Third, for synchronous chemical synaptic inputs, the waveforms resembled typical postsynaptic potentials. Whereas, for gap junctional inputs, the waveforms showed characteristics of postsynaptic potentials or dendritic spikes (accounting the active nature of inputs from the potential presynaptic cells). Electrical response of postsynaptic cell remains identical, producing an action potential regardless of whether inputs arrive via gap junctions or chemical synapses. We quantitatively matched the strengths such that the model generated a single action potential in response to synchronous inputs, irrespective of whether they arrived through chemical synaptic or gap junctional inputs. We mechanistically analyzed the contributions of different cellular components and show that the direct transmembrane current in chemical synapses is the distinguishing factor that determines the dichotomy between the contributions of gap junctions vs. chemical synapses to extracellular potentials (Fig. 23).

Fourth, for random inputs, the models were not specifically tuned to generate a single action potential. Here, the inputs served as a proxy for asynchronous inputs arriving from other subregions at random times.

Finally, the intracellular responses were comparable for chemical synaptic and gap junctional rhythmic inputs (Supplementary Fig. S5-S11). Here, the model was tuned to elicit a single spike per cycle in simulations evaluating spike-LFP interactions, ensuring a fair comparison between LFPs from gap junctional and chemical synaptic inputs.”

We have added a new Supplementary Figure 3 to the revised manuscript and have referred to this figure in the Results subsection. We thank you for raising these points as it allowed to elaborate on the several novelties and implications of our methodology and conclusions. Thank you.

Some of the explanations offered for the effects of cellular manipulations on the LFP appear to be incomplete. More specifically, the authors observed that blocking leak channels significantly changed the shape of the LFP response to synchronous synaptic inputs - but only when electric inputs were used, and when sodium channels were intact. The authors seemed to attribute this phenomenon to a direct effect of leak currents on the extracellular potential - however, this appears unlikely both because it does not explain why blocking the leak conductance had no effect in the other cases, and because the leak current is several orders of magnitude smaller than the spike-generating currents that make the largest contributions to the LFP. An indirect effect mediated by interactions of the leak current with some voltage-gated currents appears to be the most likely explanation, but identifying the exact mechanism would require further simulation experiments and/or a detailed analysis of intracellular currents and the membrane potential in time and space.

We thank you for raising this important question. Leak channels were among the several contributors to the positive deflection observed in LFPs associated with gap junctions. This effect was present not only in gap junctional models with intact sodium conductance but also in the no-sodium model, where the amplitude of the positive deflection was reduced across other models as well (Fig. 2F, I). Furthermore, even in the absence of leak conductance, a small positive deflection was still observed (Fig. 2F), leading us to further investigate other transmembrane currents over time and across spatial locations, from the proximal to the distal dendritic ends relative to the soma (Fig. 3D). We had observed that the dominant contributor in the case of chemical synapses was the inward synaptic current (Fig. 3A), whereas for gap junctions, the primary contributors were leak conductance along with other outward currents, such as potassium and HCN currents (Fig. 3D). Together, the direct transmembrane component of chemical synapses provides a dominant contribution to extracellular potentials. This dominance translates to differences in the relative contributions of indirect currents (including leak currents) to extracellular potentials associated chemical synaptic vs. gap junctional inputs. Our analyses of the exact ionic mechanisms (Fig. 3) demonstrates the involvement of several ion channels contributing to the indirect component in either scenario.

In every simulation experiment in this study, inputs through electric synapses are modeled as intracellular current injections of pre-determined amplitude and time course based on the sampled dendritic voltage of potential synaptic partners. This is a major simplification that may have a significant impact on the results. First, the current through gap junctions depends on the voltage difference between the two connected cellular compartments and is thus sensitive to the membrane potential of the cell that is treated as the neuron "receiving" the input in this study (although, strictly speaking, there is no pre- or postsynaptic neuron in interactions mediated by gap junctions). This dependence on the membrane potential of the target neuron is completely missing here. A related second point is that gap junctions also change the apparent membrane resistance of the neurons they connect, effectively acting as additional shunting (or leak) conductance in the relevant compartments. This effect is completely missed by treating gap junctions as pure current sources.

We thank you for raising this important point. We agree with the analyses presented by the reviewer on the importance of network simulations and bidirectional gap junctions that respect the voltages in both neurons. However, the complexities of LFP modeling precludes modeling of networks of morphologically realistic models with patterns of stimulations occurring across the dendritic tree. LFP modeling studies predominantly uses “post-synaptic” currents to analyze the impact of different patterns of inputs arriving on to a neuron, even when chemical synaptic inputs are considered. Explicitly, individual neurons are separately simulated with different patterns of synaptic inputs, the transmembrane current at different locations recorded, and the extracellular potential is then computed using line source approximation (Buzsaki et al., 2012; Gold et al., 2006; Halnes et al., 2024; Ness et al., 2018; Reimann et al., 2013; Schomburg et al., 2012; Sinha & Narayanan, 2015, 2022). Even in scenarios where a network is analyzed, a hybrid approach involving the outputs of a pointneuron-based network being coupled to an independent morphologically realistic neuronal model is employed (Hagen et al., 2016; Martinez-Canada et al., 2021; Mazzoni et al., 2015). Given the complexities associated with the computation of electrode potentials arising as a distance-weighted summation of several transmembrane currents, these simplifications becomes essential.

Our approach models gap junctional currents in a similar way as the other model incorporate synaptic currents in LFP modeling (Buzsaki et al., 2012; Gold et al., 2006; Halnes et al., 2024; Ness et al., 2018; Reimann et al., 2013; Schomburg et al., 2012; Sinha & Narayanan, 2015, 2022). As gap junctions are typically implemented as resistors from the other neuronal compartment, we accounted for gap-junctional variability in our model by randomizing the scaling-factors and the exact waveforms that arrive through individual gap junctions at specific locations. Thus, the inputs were not pre-determined by “pre” neurons. Instead, the recorded voltages from potential synaptic partner neurons were randomized across locations and scaled using factors at the dendrites before being injected into the target neuron (Supplementary Fig. S1). While incorporating a network of interconnected neurons is indeed important, we utilized biophysical, morphologically realistic CA1 neuron model with different sets of input patterns to model LFPs, which were derived from the total transmembrane currents across all compartments of the multi-compartmental neuron model. Given the complexity of this approach, adding further network-level interactions or pre-post connections would have been computationally demanding.

In the revised manuscript, we have elaborated on the general methodology used in LFP modeling studies to introduce synaptic currents. We have emphasized that our study extends this approach to modeling gap junctional inputs, while also highlighting randomization of locations and the scaling process in assigning gap junctional synaptic strengths. The following paragraphs were specifically added to the revised version of the manuscript:

Methods subsection “Chemical synaptic and gap junctional inputs: Characteristics and temporal dynamics”:

“The complexities of LFP modeling precludes modeling of networks of morphologically realistic models with patterns of stimulations occurring across the dendritic tree. LFP modeling studies predominantly uses post-synaptic currents to analyze the impact of different patterns of inputs arriving on to a neuron, even when chemical synaptic inputs are considered. Explicitly, individual neurons are separately simulated with different patterns of synaptic inputs, the transmembrane current at different locations recorded, and the extracellular potential is then computed using line source approximation (Buzsaki et al., 2012; Gold et al., 2006; Halnes et al., 2024; Ness et al., 2018; Reimann et al., 2013; Schomburg et al., 2012; Sinha & Narayanan, 2015, 2022). Even in scenarios where a network is analyzed, a hybrid approach involving the outputs of a point-neuron-based network being coupled to an independent morphologically realistic neuronal model is employed (Hagen et al., 2016; MartinezCanada et al., 2021; Mazzoni et al., 2015). Given the complexities associated with the computation of electrode potentials arising as a distance-weighted summation of several transmembrane currents, these simplifications become essential.”

“Our approach models gap junctional currents in a similar way as the other model incorporate synaptic currents in LFP modeling (Buzsaki et al., 2012; Gold et al., 2006; Halnes et al., 2024; Ness et al., 2018; Reimann et al., 2013; Schomburg et al., 2012; Sinha & Narayanan, 2015, 2022). As gap junctions are typically implemented as resistors from the other neuronal compartment, we accounted for gap-junctional variability in our model by randomizing the scaling-factors and the exact waveforms that arrive through individual gap junctions at specific locations from potential presynaptic sources.”

We thank for you highlighting these points as it allowed us to place our methodology in the specific context of the literature. Thank you.

One prominent claim of the article that is emphasized even in the abstract is that HCN channels mediate an outward current in certain cases. Although this statement is technically correct, there are two reasons why I do not consider this a major finding of the paper. First, as the authors acknowledge, this is a trivial consequence of the relatively slow kinetics of HCN channels: when at least some of the channels are open, any input that is sufficiently fast and strong to take the membrane potential across the reversal potential of the channel will lead to the reversal of the polarity of the current. This effect is quite generic and well-known and is by no means specific to gap junctional inputs or even HCN channels. Second, and perhaps more importantly, the functional consequence of this reversed current through HCN channels is likely to be negligible. As clearly shown in Supplementary Figure S3, the HCN current becomes outward only for an extremely short time period during the action potential, which is also a period when several other currents are also active and likely dominant due to their much higher conductances. I also note that several of these relevant facts remain hidden in Figure 3, both because of its focus on peak values, and because of the radically different units on the vertical axes of the current plots.

We thank you for raising this point and agree with you on every point. Please note that we do not assert that the outward HCN currents are exclusively associated with gap junctional inputs. Rather, our results show that synchronous inputs generate outward HCN currents in both chemical synapses (Fig. 3B; positive/outward HCN currents, except in the no sodium or leak model) and gap junctions (Fig. 3D; positive/outward HCN currents). We emphasized this in the case of gap junctions because, in the absence of inward synaptic currents, HCN (acting as outward currents with synchronous inputs) contributed to the positive deflection observed in the LFPs. While HCN would also contribute in the case of chemical synapses, its effect was negligible due to the presence of large inward synaptic currents. Since LFPs reflect the collective total transmembrane currents, the dominant contributors differ between these two scenarios, which we aimed to highlight. Since HCN exhibited outward currents in our synchronous input simulations, we have elaborated on this mechanism in the supplementary figure (Fig. S3). Our intention was not to emphasize this effect for only one synaptic mode but rather to highlight HCN's contribution to the positive deflection as one of the contributing factors.

We agree that HCN currents are relatively small in magnitude; therefore, our conclusions were based on HCN being one of the several contributing factors. Leak conductance and other outward conductances, including HCN currents (Fig. 3D), collectively contribute to the positive deflections observed in the case of gap junctional synchronous inputs.

In the revised manuscript, we have provided the following clarifications in the Results subsection on” Synchronous inputs: Outward transmembrane currents from active dendrites contribute to positive deflection in extracellular potentials associated with gap junctional inputs”:

“It is important to note that despite their relatively small magnitude, the outward HCN currents (Fig. 3D) substantially contribute to positive extracellular potential deflections associated with gap junctional inputs (Fig. 2), together with leak and other outward conductances.”

“While outward HCN currents (Fig. 3B) are also expected to influence LFPs under chemical synaptic input, their impact was minimal due to the predominance of large inward synaptic currents (Fig. 3A). As LFPs reflect the summation of all transmembrane currents, the dominant contributors vary across different modes of synaptic transmission.”

We thank you for emphasizing this point. This allowed us to expand on the specific roles of HCN channels and potential contributions of the outward nature of the HCN current. We have also expanded the Discussion subsection on “Outward HCN currents regulate extracellular potentials” to elaborate on this aspect as well. Thank you.

Finally, I missed an appropriate validation of the neuronal model used, and also the characterization of the effects of the in silico manipulations used on the basic behavior of the model. As far as I understand, the model in its current form has not been used in other studies. If this is the case, it would be important to demonstrate convincingly through (preferably quantitative) comparisons with experimental data using different protocols that the model captures the physiological behavior of at least the relevant compartments (in this case, the dendrites and the soma) of hippocampal pyramidal neurons sufficiently well that the results of the modeling study are relevant to the real biological system. In addition, the correct interpretation of various manipulations of the model would be strongly facilitated by investigating and discussing how the physiological properties of the model neuron are affected by these alterations.

We thank you for raising this important point. The CA1 pyramidal neuronal model used in this study is built with ion-channel models derived from biophysical and electrophysiological recordings from these cells. As mentioned in the Methods section “Dynamics and distribution of active channels” and Supplementary Table S1, models for individual channels, their gating kinetics, and channel distributions across the somatodendritic arbor (wherever known) are all derived from their physiological equivalents. Importantly, these values were derived from previously validated models from the laboratory, which contain these very ion channel models and the exact same morphology (Roy & Narayanan, 2021). Please compare Supplementary Table S1 with Table 1 from (Roy & Narayanan, 2021). Please note that this model was validated against several physiological measurements along the somatodendritic axis (Fig. 1 of (Roy & Narayanan, 2021)).

In the revised manuscript, we have explicitly mentioned this while also mentioning the different physiological properties that were used for the validation process from (Roy & Narayanan, 2021):

Methods subsection “Pyramidal neuron model”

“All parameters and their corresponding values for the neuronal model were derived from previously validated models (Roy & Narayanan, 2021). These CA1 models were validated against several physiological measurements along the somato dendritic axis (Roy & Narayanan, 2021).”

“These channel distributions and the associated parametric values (Supplementary Table S1) were demonstrated to satisfy 22 different somato-dendritic measurements (Roy & Narayanan, 2021). Specifically, six physiological measurements input resistance, maximal impedance amplitude, resonance frequency, resonance strength, total inductive phase, and back-propagating action potential were validated with respective electrophysiological ranges at three somato-dendritic locations (Soma, ~150 µm dendrite, and ~300 µm dendrite) each (6×3=18 measurements). In addition, action potential firing frequency at each of 100 pA, 150 pA, 200 pA, and 250 pA (4 measurements) were also matched in the model to fall within the respective ranges of corresponding electrophysiological measurements. The electrophysiological ranges of intrinsic measurements were derived from respective somato-dendritic recordings (Malik et al., 2016; Narayanan et al., 2010; Narayanan & Johnston, 2007, 2008; Spruston et al., 1995). Together, the CA1 pyramidal model neuron used in this study was tuned to match several electrophysiological characteristics and ion-channel distributions (Roy & Narayanan, 2021).”

We thank you for pointing us to this slip in elaborating on how the model was validated. We have now rectified this. Thank you.

References

Andersen, P., Morris, R., Amaral, D., Bliss, T., & O'Keefe, J. (2006). The hippocampus book. Oxford University Press.

Basak, R., & Narayanan, R. (2018). Spatially dispersed synapses yield sharply-tuned place cell responses through dendritic spike initiation. Journal of Physiology, 596(17), 4173-4205. https://doi.org/10.1113/JP275310

Bedner, P., Steinhauser, C., & Theis, M. (2012). Functional redundancy and compensation among members of gap junction protein families? Biochim Biophys Acta, 1818(8), 1971-1984. https://doi.org/10.1016/j.bbamem.2011.10.016

Behrens, C. J., Ul Haq, R., Liotta, A., Anderson, M. L., & Heinemann, U. (2011). Nonspecific effects of the gap junction blocker mefloquine on fast hippocampal network oscillations in the adult rat in vitro. Neuroscience, 192, 11-19. https://doi.org/10.1016/j.neuroscience.2011.07.015

Bocian, R., Posluszny, A., Kowalczyk, T., Golebiewski, H., & Konopacki, J. (2009). The effect of carbenoxolone on hippocampal formation theta rhythm in rats: in vitro and in vivo approaches. Brain Res Bull, 78(6), 290-298. https://doi.org/10.1016/j.brainresbull.2008.10.005

Buhl, D. L., Harris, K. D., Hormuzdi, S. G., Monyer, H., & Buzsaki, G. (2003). Selective impairment of hippocampal gamma oscillations in connexin-36 knock-out mouse in vivo. J Neurosci, 23(3), 1013-1018. https://doi.org/10.1523/jneurosci.23-03-01013.2003

Buzsaki, G., Anastassiou, C. A., & Koch, C. (2012). The origin of extracellular fields and currents--EEG, ECoG, LFP and spikes. Nat Rev Neurosci, 13(6), 407-420. https://doi.org/10.1038/nrn3241

Buzsaki, G., & Wang, X. J. (2012). Mechanisms of gamma oscillations. Annual Review of Neuroscience, Vol 36, 35, 203-225. https://doi.org/10.1146/annurev-neuro-062111150444

Colgin, L. L. (2016). Rhythms of the hippocampal network. Nat Rev Neurosci, 17(4), 239249. https://doi.org/10.1038/nrn.2016.21

Colgin, L. L., & Moser, E. I. (2010). Gamma oscillations in the hippocampus. Physiology (Bethesda), 25(5), 319-329. https://doi.org/10.1152/physiol.00021.2010

Coulon, P., & Landisman, C. E. (2017). The Potential Role of Gap Junctional Plasticity in the Regulation of State. Neuron, 93(6), 1275-1295. https://doi.org/10.1016/j.neuron.2017.02.041

Das, A., Rathour, R. K., & Narayanan, R. (2017). Strings on a Violin: Location Dependence of Frequency Tuning in Active Dendrites. Front Cell Neurosci, 11, 72. https://doi.org/10.3389/fncel.2017.00072

Draguhn, A., Traub, R. D., Schmitz, D., & Jefferys, J. G. (1998). Electrical coupling underlies high-frequency oscillations in the hippocampus in vitro. Nature, 394(6689), 189-192. https://doi.org/10.1038/28184

Einevoll, G. T., Destexhe, A., Diesmann, M., Grun, S., Jirsa, V., de Kamps, M., Migliore, M., Ness, T. V., Plesser, H. E., & Schurmann, F. (2019). The Scientific Case for Brain Simulations. Neuron, 102(4), 735-744. https://doi.org/10.1016/j.neuron.2019.03.027

Einevoll, G. T., Kayser, C., Logothetis, N. K., & Panzeri, S. (2013). Modelling and analysis of local field potentials for studying the function of cortical circuits. Nat Rev Neurosci, 14(11), 770-785. https://doi.org/10.1038/nrn3599

Gold, C., Henze, D. A., Koch, C., & Buzsaki, G. (2006). On the origin of the extracellular action potential waveform: A modeling study. J Neurophysiol, 95(5), 3113-3128. https://doi.org/10.1152/jn.00979.2005

Hagen, E., Dahmen, D., Stavrinou, M. L., Linden, H., Tetzlaff, T., van Albada, S. J., Grun, S., Diesmann, M., & Einevoll, G. T. (2016). Hybrid Scheme for Modeling Local Field Potentials from Point-Neuron Networks. Cereb Cortex, 26(12), 4461-4496. https://doi.org/10.1093/cercor/bhw237

Halnes, G., Ness, T. V., Næss, S., Hagen, E., Pettersen, K. H., & Einevoll, G. T. (2024). Electric Brain Signals: Foundations and Applications of Biophysical Modeling. Cambridge University Press. https://doi.org/10.1017/9781009039826

Hormuzdi, S. G., Pais, I., LeBeau, F. E., Towers, S. K., Rozov, A., Buhl, E. H., Whittington, M. A., & Monyer, H. (2001). Impaired electrical signaling disrupts gamma frequency oscillations in connexin 36-deficient mice. Neuron, 31(3), 487-495. https://doi.org/10.1016/s0896-6273(01)00387-7

Hussaini, S. A., Kempadoo, K. A., Thuault, S. J., Siegelbaum, S. A., & Kandel, E. R. (2011). Increased size and stability of CA1 and CA3 place fields in HCN1 knockout mice. Neuron, 72(4), 643-653. https://doi.org/10.1016/j.neuron.2011.09.007

Johnston, D., & Narayanan, R. (2008). Active dendrites: colorful wings of the mysterious butterflies. Trends Neurosci, 31(6), 309-316. https://doi.org/10.1016/j.tins.2008.03.004

Kessi, M., Peng, J., Duan, H., He, H., Chen, B., Xiong, J., Wang, Y., Yang, L., Wang, G., Kiprotich, K., Bamgbade, O. A., He, F., & Yin, F. (2022). The Contribution of HCN Channelopathies in Different Epileptic Syndromes, Mechanisms, Modulators, and Potential Treatment Targets: A Systematic Review. Front Mol Neurosci, 15, 807202. https://doi.org/10.3389/fnmol.2022.807202

Kole, M. H., Hallermann, S., & Stuart, G. J. (2006). Single Ih channels in pyramidal neuron dendrites: properties, distribution, and impact on action potential output [Research Support, Non-U.S. Gov't]. J Neurosci, 26(6), 1677-1687. https://doi.org/10.1523/JNEUROSCI.3664-05.2006

Konopacki, J., Kowalczyk, T., & Golebiewski, H. (2004). Electrical coupling underlies theta oscillations recorded in hippocampal formation slices. Brain Res, 1019(1-2), 270-274. https://doi.org/10.1016/j.brainres.2004.05.083

Larkum, M. E., Wu, J., Duverdin, S. A., & Gidon, A. (2022). The Guide to Dendritic Spikes of the Mammalian Cortex In Vitro and In Vivo. Neuroscience, 489, 15-33. https://doi.org/10.1016/j.neuroscience.2022.02.009

LeBeau, F. E., Traub, R. D., Monyer, H., Whittington, M. A., & Buhl, E. H. (2003). The role of electrical signaling via gap junctions in the generation of fast network oscillations. Brain Res Bull, 62(1), 3-13. https://doi.org/10.1016/j.brainresbull.2003.07.004

Lo, C. W. (1999). Genes, gene knockouts, and mutations in the analysis of gap junctions. Dev Genet, 24(1-2), 1-4. https://doi.org/10.1002/(SICI)1520-6408(1999)24:1/2%3C1::AID-DVG1%3E3.0.CO;2-U

Lorincz, A., Notomi, T., Tamas, G., Shigemoto, R., & Nusser, Z. (2002). Polarized and compartment-dependent distribution of HCN1 in pyramidal cell dendrites. Nat Neurosci, 5(11), 1185-1193. https://doi.org/10.1038/nn962

Magee, J. C. (1998). Dendritic hyperpolarization-activated currents modify the integrative properties of hippocampal CA1 pyramidal neurons. J Neurosci, 18(19), 7613-7624. https://doi.org/10.1523/jneurosci.18-19-07613.1998

Magee, J. C., & Grienberger, C. (2020). Synaptic Plasticity Forms and Functions. Annual Review of Neuroscience, Vol 36, 43, 95-117. https://doi.org/10.1146/annurev-neuro090919-022842

Major, G., Larkum, M. E., & Schiller, J. (2013). Active properties of neocortical pyramidal neuron dendrites [Review]. Annual Review of Neuroscience, Vol 36, 36, 1-24. https://doi.org/10.1146/annurev-neuro-062111-150343

Malik, R., Dougherty, K. A., Parikh, K., Byrne, C., & Johnston, D. (2016). Mapping the electrophysiological and morphological properties of CA1 pyramidal neurons along the longitudinal hippocampal axis. Hippocampus, 26(3), 341-361. https://doi.org/10.1002/hipo.22526

Martinez-Canada, P., Ness, T. V., Einevoll, G. T., Fellin, T., & Panzeri, S. (2021). Computation of the electroencephalogram (EEG) from network models of point neurons. PLoS Comput Biol, 17(4), e1008893. https://doi.org/10.1371/journal.pcbi.1008893

Mazzoni, A., Linden, H., Cuntz, H., Lansner, A., Panzeri, S., & Einevoll, G. T. (2015). Computing the Local Field Potential (LFP) from Integrate-and-Fire Network Models. PLoS Comput Biol, 11(12), e1004584. https://doi.org/10.1371/journal.pcbi.1004584

Mishra, P., & Narayanan, R. (2021). Stable continual learning through structured multiscale plasticity manifolds. Curr Opin Neurobiol, 70, 51-63. https://doi.org/10.1016/j.conb.2021.07.009

Mishra, P., & Narayanan, R. (2025). The enigmatic HCN channels: A cellular neurophysiology perspective. Proteins, 93(1), 72-92. https://doi.org/10.1002/prot.26643

Moore, J. J., Ravassard, P. M., Ho, D., Acharya, L., Kees, A. L., Vuong, C., & Mehta, M. R. (2017). Dynamics of cortical dendritic membrane potential and spikes in freely behaving rats. Science, 355(6331). https://doi.org/10.1126/science.aaj1497

Narayanan, R., Dougherty, K. J., & Johnston, D. (2010). Calcium Store Depletion Induces Persistent Perisomatic Increases in the Functional Density of h Channels in Hippocampal Pyramidal Neurons. Neuron, 68(5), 921-935. https://doi.org/10.1016/j.neuron.2010.11.033

Narayanan, R., & Johnston, D. (2007). Long-term potentiation in rat hippocampal neurons is accompanied by spatially widespread changes in intrinsic oscillatory dynamics and excitability. Neuron, 56(6), 1061-1075. https://doi.org/10.1016/j.neuron.2007.10.033

Narayanan, R., & Johnston, D. (2008). The h channel mediates location dependence and plasticity of intrinsic phase response in rat hippocampal neurons. J Neurosci, 28(22), 5846-5860. https://doi.org/10.1523/JNEUROSCI.0835-08.2008

Ness, T. V., Remme, M. W. H., & Einevoll, G. T. (2016). Active subthreshold dendritic conductances shape the local field potential. Journal of Physiology, 594(13), 38093825. https://doi.org/10.1113/JP272022

Ness, T. V., Remme, M. W. H., & Einevoll, G. T. (2018). h-Type Membrane Current Shapes the Local Field Potential from Populations of Pyramidal Neurons. J Neurosci, 38(26), 6011-6024. https://doi.org/10.1523/jneurosci.3278-17.2018

Neves, G., Cooke, S. F., & Bliss, T. V. (2008). Synaptic plasticity, memory and the hippocampus: a neural network approach to causality. Nat Rev Neurosci, 9(1), 65-75. https://doi.org/10.1038/nrn2303

Nolan, M. F., Malleret, G., Dudman, J. T., Buhl, D. L., Santoro, B., Gibbs, E., Vronskaya, S., Buzsaki, G., Siegelbaum, S. A., Kandel, E. R., & Morozov, A. (2004). A behavioral role for dendritic integration: HCN1 channels constrain spatial memory and plasticity at inputs to distal dendrites of CA1 pyramidal neurons. Cell, 119(5), 719-732. https://doi.org/10.1016/j.cell.2004.11.020

O'Brien, J. (2014). The ever-changing electrical synapse. Curr Opin Neurobiol, 29, 64-72. https://doi.org/10.1016/j.conb.2014.05.011

O'Keefe, J., & Recce, M. L. (1993). Phase relationship between hippocampal place units and the EEG theta rhythm. Hippocampus, 3(3), 317-330. https://doi.org/10.1002/hipo.450030307

Pereda, A. E. (2014). Electrical synapses and their functional interactions with chemical synapses. Nat Rev Neurosci, 15(4), 250-263. https://doi.org/10.1038/nrn3708

Posluszny, A. (2014). The contribution of electrical synapses to field potential oscillations in the hippocampal formation. Front Neural Circuits, 8, 32. https://doi.org/10.3389/fncir.2014.00032

Reimann, M. W., Anastassiou, C. A., Perin, R., Hill, S. L., Markram, H., & Koch, C. (2013). A biophysically detailed model of neocortical local field potentials predicts the critical role of active membrane currents. Neuron, 79(2), 375-390. https://doi.org/10.1016/j.neuron.2013.05.023

Rouach, N., Segal, M., Koulakoff, A., Giaume, C., & Avignone, E. (2003). Carbenoxolone blockade of neuronal network activity in culture is not mediated by an action on gap junctions. Journal of Physiology, 553(Pt 3), 729-745. https://doi.org/10.1113/jphysiol.2003.053439

Roy, A., & Narayanan, R. (2021). Spatial information transfer in hippocampal place cells depends on trial-to-trial variability, symmetry of place-field firing, and biophysical heterogeneities. Neural Netw, 142, 636-660. https://doi.org/10.1016/j.neunet.2021.07.026

Schomburg, E. W., Anastassiou, C. A., Buzsaki, G., & Koch, C. (2012). The spiking component of oscillatory extracellular potentials in the rat hippocampus. J Neurosci, 32(34), 11798-11811. https://doi.org/10.1523/JNEUROSCI.0656-12.2012

Seenivasan, P., & Narayanan, R. (2020). Efficient phase coding in hippocampal place cells. Physical Review Research, 2(3), 033393. https://doi.org/10.1103/PhysRevResearch.2.033393

Sinha, M., & Narayanan, R. (2015). HCN channels enhance spike phase coherence and regulate the phase of spikes and LFPs in the theta-frequency range. Proc Natl Acad Sci U S A, 112(17), E2207-2216. https://doi.org/10.1073/pnas.1419017112

Sinha, M., & Narayanan, R. (2022). Active Dendrites and Local Field Potentials: Biophysical Mechanisms and Computational Explorations. Neuroscience, 489, 111-142. https://doi.org/10.1016/j.neuroscience.2021.08.035

Sirmaur, R., & Narayanan, R. (2024). Distinct extracellular signatures of chemical and electrical synapses impinging on active dendrites differentially contribute to ripplefrequency oscillations. Society for Neuroscience annual meeting, Chicago, USA.

Spruston, N., Schiller, Y., Stuart, G., & Sakmann, B. (1995). Activity-dependent action potential invasion and calcium influx into hippocampal CA1 dendrites [Research Support, Non-U.S. Gov't]. Science, 268(5208), 297-300. https://doi.org/10.1126/science.7716524

Stuart, G. J., & Spruston, N. (2015). Dendritic integration: 60 years of progress. Nat Neurosci, 18(12), 1713-1721. https://doi.org/10.1038/nn.4157

Szarka, G., Balogh, M., Tengolics, A. J., Ganczer, A., Volgyi, B., & Kovacs-Oller, T. (2021). The role of gap junctions in cell death and neuromodulation in the retina. Neural Regen Res, 16(10), 1911-1920. https://doi.org/10.4103/1673-5374.308069

Traub, R. D., Cunningham, M. O., Gloveli, T., LeBeau, F. E., Bibbig, A., Buhl, E. H., & Whittington, M. A. (2003). GABA-enhanced collective behavior in neuronal axons underlies persistent gamma-frequency oscillations. Proc Natl Acad Sci U S A, 100(19), 11047-11052. https://doi.org/10.1073/pnas.1934854100

Vaughn, M. J., & Haas, J. S. (2022). On the Diverse Functions of Electrical Synapses. Front Cell Neurosci, 16, 910015. https://doi.org/10.3389/fncel.2022.910015

Wang, X. J. (2010). Neurophysiological and computational principles of cortical rhythms in cognition. Physiol Rev, 90(3), 1195-1268. https://doi.org/10.1152/physrev.00035.2008

Wang, X. J., & Buzsaki, G. (1996). Gamma oscillation by synaptic inhibition in a hippocampal interneuronal network model. J Neurosci, 16(20), 6402-6413. https://doi.org/10.1523/jneurosci.16-20-06402.1996

Whittington, M. A., Traub, R. D., & Jefferys, J. G. (1995). Synchronized oscillations in interneuron networks driven by metabotropic glutamate receptor activation. Nature, 373(6515), 612-615. https://doi.org/10.1038/373612a0

Williams, S. R., & Stuart, G. J. (2000). Site independence of EPSP time course is mediated by dendritic I(h) in neocortical pyramidal neurons [In Vitro]. J Neurophysiol, 83(5), 3177-3182. https://doi.org/10.1152/jn.2000.83.5.3177
